# Mapping of soil suitability for medicinal plants using machine learning methods

**DOI:** 10.1038/s41598-024-54465-3

**Published:** 2024-02-14

**Authors:** S. Roopashree, J. Anitha, Suryateja Challa, T. R. Mahesh, Vinoth Kumar Venkatesan, Suresh Guluwadi

**Affiliations:** 1 Department of Computer Science and Engineering, RV Institute of Technology and Management, Bengaluru, Karnataka India; 2 Department of Computer Science and Engineering, JAIN (Deemed-to-be University), Bengaluru, Karnataka India; 3 School of Computer Science Engineering & Information Systems (SCORE), Vellore Institute of Technology (VIT), Vellore, 632014 India; 4 Adama Science and Technology University, 302120 Adama, Ethiopia

**Keywords:** Choropleth, Decision tree, Extra trees classifier, Geographic information system, Machine learning, Medicinal plants, Supervised learning, Computational biology and bioinformatics, Plant sciences, Environmental social sciences, Health care, Molecular medicine, Engineering

## Abstract

Inadequate conservation of medicinal plants can affect their productivity. Traditional assessments and strategies are often time-consuming and linked with errors. Utilizing herbs has been an integral part of the traditional system of medicine for centuries. However, its sustainability and conservation are critical due to climate change, over-harvesting and habitat loss. The study reveals how machine learning algorithms, geographic information systems (GIS) being a powerful tool for mapping and spatial analysis, and soil information can contribute to a swift decision-making approach for actual forethought and intensify the productivity of vulnerable curative plants of specific regions to promote drug discovery. The data analysis based on machine learning and data mining techniques over the soil, medicinal plants and GIS information can predict quick and effective results on a map to nurture the growth of the herbs. The work incorporates the construction of a novel dataset by using the quantum geographic information system tool and recommends the vulnerable herbs by implementing different supervised algorithms such as extra tree classifier (EXTC), random forest, bagging classifier, extreme gradient boosting and k nearest neighbor. Two unique approaches suggested for the user by using EXTC, firstly, for a given subregion type, its suitable soil classes and secondly, for soil type from the user, its respective subregion labels are revealed, finally, potential medicinal herbs and their conservation status are visualised using the choropleth map for classified soil/subregion. The research concludes on EXTC as it showcases outstanding performance for both soil and subregion classifications compared to other models, with an accuracy rate of 99.01% and 98.76%, respectively. The approach focuses on serving as a comprehensive and swift reference for the general public, bioscience researchers, and conservationists interested in conserving medicinal herbs based on soil availability or specific regions through maps.

## Introduction

Endangered medicinal plants face the imminent risk of extinction due to many factors, including over-harvesting, habitat loss, climate change, invasive species, diseases, and pests. These plants, deeply rooted in traditional systems of Indian medicine like Ayurveda, Siddha, Unani, and Tibetan medicine, hold immense value in both the traditional and modern pharmaceuticals. They provide significant health benefits and offer income opportunities to millions of people reliant on them. Examples of such endangered plants include *Saussaurea lappa (Kuth), Picorrhiza kurroa (Kutki), Ginkgo biloba (Maidenhair tree), Swertia chirata (Chirayata), Gymnema sylvestre (Gurmar), Tinospora cordifolia (Giloy), Salaca oblonga (Salak), Holostemma (Jivanti), Celastrus paniculata (Malkangni), Oroxylum indicum (Shyonaka), Glycyrrhiza glabra (Licorice), Tylophora indica (Antamul), Bacopa monnieri (Brahmi), and Rauwolfia serpentina (Sarpagandha).* These plants possess various medicinal properties, ranging from anti-inflammatory, anti-diabetic, anti-microbial, anti-cancer, neuroprotective, hepatoprotective, and immunomodulatory effects. Additionally, they serve as valuable sources of compounds used in pharmaceuticals, such as *reserpine* from *Snakeroot* and *paclitaxel* from *Himalayan Yew*. Furthermore, several of these plants are esteemed for their aromatic or timber qualities, exemplified by *Jatamansi oil* and *Red Sanders wood*^1^.

The collection of these plants predominantly occurs in the wild, particularly within the alpine regions of the Himalayas, where they endure harsh conditions and exhibit slow regeneration rates. The demand for these plants has recently surged in domestic and foreign markets due to the growing popularity of natural and herbal products. Consequently, unsustainable harvesting practices and over-exploitation pose severe threats to the survival of these plants within their natural habitats. According to a recent report by the IUCN (The International Union for Conservation of Nature) and TRAFFIC (a registered non-governmental UK charity)^2^, India is a hub for the wild-collected plant medicine industry in Asia. However, several vital species have experienced notable declines due to excessive collection to meet the demands of domestic and foreign medicinal markets.

Conservation efforts^3^ targeting these plants hold paramount importance for the well-being and livelihoods of millions reliant on their medicinal properties. Furthermore, conserving these plants is vital for preserving biodiversity and sustaining ecosystem services. Nonetheless, various challenges impede effective conservation initiatives, including inadequate data on species status and distribution, inadequate legal and policy frameworks, sub-optimal implementation and enforcement of existing regulations, insufficient involvement and awareness among local communities and stakeholders, limited funding and technical support, and competing interests in land use. Therefore, urgent collaborative actions among diverse stakeholders such as government agencies, research institutions, conservation organisations, industry associations, traders, collectors, healers, consumers, and the media must ensure sustainable management and utilisation of these plants.

Machine learning (ML) actively contributes to the growth of medicinal plants in various parameters. Few are in the process of enhancing the yield, quality, and diversity of plant biomass and metabolites. Multiple factors, including genetics, environment, and biotics, influence the growth of medicinal plants. ML techniques can assist in analysing the above factors and optimise cultivation conditions and practices for medicinal plants. For example, ML possess the ability to predict the optimal harvesting time, irrigation schedule, fertilisation rate, pest control strategy, and post-harvest processing for medicinal plants based on their phenotypic and physiological traits^4^. ML algorithms can determine the optimum plant kinds or genotypes for various climatic zones and soil types based on their genetic and molecular markers. A systematic review reveals how different ML techniques can select the traits in prime crops^5^. Additionally, it can categorise medicinal plants based on the characteristics of their leaves, such as colour, vein, and shape, using its digital images. The application of deep learning concepts highly supports to recognise the medicinal plants of India^6^. The crops such as winter wheat and garlic are identified based on multi-source remote sensing information^7^.

Machine learning techniques coupled with GIS (Geographic Information System) tools, such as Quantum Geographic Information System (QGIS), can highly assist in the growth of medicinal plants in specific regions by considering geospatial data and soil types^8^. Geospatial data plays a crucial role in understanding the spatial patterns and relationships between different variables in a given area, identifying suitable regions for cultivating specific medicinal plants, and implementing targeted management strategies^9^. The GIS tool provides a robust framework for organising, analysing, and visualising the geospatial data. The scientists reveal that QGIS, an open-source tool, allows for integrating and visualising diverse data types. By adding layers using shapefiles in QGIS, researchers can easily incorporate multiple datasets and perform spatial analysis on the distribution of medicinal plants^10^. The researchers^11^ discuss on developing a GMPGIS (Global Medicinal Plant Geographic Information System) to analyse environmental information of ecologically suitable regions, thus guiding the conservation and introduction of medicinal plants.

The knowledge of medicinal herbs, their potential uses and their silent disappearance is known to native users and not the public. It is crucial to overcome this deficiency to lead a healthy herbal life avoiding the consumption of indirect chemicals through medicines that eventually lead to side effects. However, the best approach is to rely on cost-effective medicines from herbs. The work focussed on targeting a solution to identify the potential medicinal plants (vulnerable herbs) with knowledge of their soil type and the region of interest to enhance their growth. The soil type and the suitable region associated with every medicinal herb are essential to growing herbs at high quality preserving all its medicinal properties. A comprehensive understanding of the above factors plays a vital role in enhancing its growth. The research proposes a novel combination that includes machine learning algorithms, GIS tools like QGIS, geospatial data, and soil information as a robust approach to understanding the relationship between soil textural types and the distribution of medicinal plants. By leveraging these techniques, the stakeholders can afford easy decisions on the cultivation and management of medicinal plants, ultimately supporting sustainable practices and the conservation of therapeutic plant resources. This study is crucial to concentrate on the medicinal herbs disappearing silently before their potential uses are known by their stakeholders.

The motivation for the current research stems from the domineering need to optimize the recommendation procedure to cultivate vulnerable herbs in an era where natural remedies and sustainable practices are highly valued. It is pivotal for the accurate recognition of herbs with ideal location on the map. Hence, leveraging supervised learning, GIS and soil-based analysis holds immense potential in revolutionizing the sustainable utilization, harvest and cultivation of endangered medicinal flora.

The chief contributions of the proposed methodology for the design of a recommendation model using a supervised learning (decision tree) algorithm for the growth of the vulnerable medicinal plants of a region using its soil type and GIS information as key parameters to highlight its potential production regions include: (1) Generation of the custom dataset of 160,492 rows for a specific region of interest (Karnataka, southern state of India) using QGIS by extracting the attributes such as latitude, longitude, soil type and region. (2) Construct a novel custom database concentrating on endangered medicinal plants of a selected region (India), with attributes such as their conservation status, soil type, and region name. (3) The designed approach recommends the locations and the potential medicinal plant species for a given soil type of a specific region and also recommends the soil type and the potential medicinal plant species for a given region of choice. (4) The predicted results reveal the recommended medicinal plant(s) using the choropleth map to enhance its knowledge among the stakeholders by viewing on the map.

The paper’s organisation is as follows: The related works section describes how different works contribute to identifying medicinal herbs using various techniques. The methodology section briefs on the proposed strategies to design and develop the proposed system by locating the vulnerable herbs on maps. The results and discussion section disclose the validation of results from the decision tree technique and GIS-based models on the region of interest (Karnataka region). The suggested system is compared against other related works to exhibit its efficacy. Lastly, the conclusion section unfolds the impact of the proposed approach on the growth of medicinal herbs (endangered) and their significance to the world.

## Related works

India is a country well known as the “Medical Garden” of the world^12^. India has a rich biodiversity of 9500 species with ethnobotanical notability and 7500 medicinal plant species with medicinal values for the indigenous health exercise and the modern medicine system^13,14^. From the ancient period, Indian medicinal herbs have been the chief source of raw material for India’s traditional health care system and thus provide a livelihood to India’s colossal population^15^. Various approaches are adopted in India to conserve medicinal plants by listing exclusively the vulnerable herbs. Investigators highlight the importance of providing easy access to the associated details of these herbs as it is essential to the stakeholders to nurture its growth and knowledge^16^. It is crucial to develop divergent tactics for the productive cultivation of medicinal plants and ensure their availability for future generations. The extinction of medicinal plants is rising at a higher rate when compared to general plant species. The knowledge of regrowing these medicinal herbs in suitable soil, region and other environmental factors is critical to stakeholders as the traditional approach is tedious, inefficient and error prone.

A global medicinal plant geographic information system^11^ utilizes the geographic information for the global herbs that analyse the various information on suitable regions to conserve and introduce medicinal plants by considering both soil and climate variations. They showcased the high potential regions around the world to grow herbs. The use of GIS data^17^ by extracting the shape files (GIS layers) for any area of interest and plotting the correlation between the medicinal herbs and the altitude. The GIS techniques help to recognise the threatened herb spots and the possible conservation hotspots for the northwest Jordan region by highlighting the globally important medicinal herbs. The GIS tools can be extended to different areas of interest to enhance the research study. The researchers^18^ concentrated on probable medicinal plants (*Angelica keiskei, Ecklonia cava, Torreya nucifera* and many more) that can serve as a nutraceutical to supervise COVID-19 for the public at a reduced cost. The work presents the curation of facts from multiple sources (search engines) such as Scopus, Web of Science, PubMed and Google Scholar to retrieve information on the usage of medicinal plant compounds and their inhibitory effects to cure SARS-CoV 3CL. The gathered medicinal herbs are traditionally utilized to alleviate the disease (SARS-CoV) infection, and the reported compounds are utilized to derive anti-drugs for SARS. Accordingly, there is an immediate need to conserve such medicinal plants as they are safe resources and grow them in abundance as an economical product of a country.

The researchers proposed a few preservation compensations and advantages of distinct frameworks to control the protection of varied species in nature^19^. It is essential and ongoing research to design and develop a recognition system to identify the medicinal herbs from their leaf image as it stands as the preferred choice for any recognition of plant species^20^. The scientists designed a prolific system to identify the plant species from leaf images of complex backgrounds^21^. A computationally efficient architecture to identify the plant species using leaf images was proposed^22^. Many types of features can be automatically extracted from the given input image using various image processing and machine learning techniques. The classification of medicinal herbs by developing robust classifiers assists in recognising the species in real time utilising reliable and effective ML algorithms^23^. The review presents different image processing approaches employed to extract the features of the leaf and the publicly available plant leaf databases that assist in the automatic recognition of the same. The research area for classifying and conserving medicinal plants is prominently ongoing, and there exists a scope of opportunities for its enhancement. However, an automatic classification system and locating the herb position on maps will be an optimal solution for the local population to enhance the growth of herbs.

The prediction of soil series and suggestions on suitable crop yields for a specific soil is presented^24^ for the Khulna region in Bangladesh. The adopted machine learning classifiers such as random forest, kNN (k nearest neighbors) and SVM (support vector machine) shows better accuracy in soil classification. The SVM model outperformed the other two techniques by an accuracy rate of 94.95%. The proposed system classified the selected soil labels and recommended suitable crops by merging multiple databases and suggested to consider different regions of interest as future scope. An automatic system to classify the medicinal plants of Brazil’s region adopted supervised learners such as the decision tree algorithm and random forest technique^25^ to exhibit good classification accuracy in recognising medicinal plants from their leaf image. The random forest classifier showed the highest accuracy rate and low prediction time to identify the herbs based on their unique attributes such as colour and texture features. The researchers^26^ presented the importance of nurturing the *Taxus baccata* plant with anti-cancer capabilities by incorporating the GIS and remote sensing data providing an ideal solution for recognising the region of potential growth. A work^27^ proposed an approach using QGIS as the visual analysis to manage the waste in South Korea by highlighting the optimal route for the truck carrying the waste. The suggested approach showed better performance analysis in planning and optimizing the operations of waste management. Land or soil suitability plays a significant role in identifying the potential growth spots to conserve medicinal plants as the herbs possess specific requirements^28,29^.

From the survey, it is noteworthy to highlight that there exists high scope on conserving and growing vulnerable herbs. It is evident that the Quantum GIS technique and soil information together contribute efficiently to identify the conservation and potential production spots for herbs. Employing GIS as the spatial database and soil information derived from QGIS can be a systematic approach to nurture the growth of vulnerable medicinal plants. An automatic user-friendly system amalgamating the above techniques with machine learning algorithms proves to be critical for the medicinal plants listed in the IUCN red list before they reach the state of extinction.

## Methodology

### Study area

**Figure 1:**
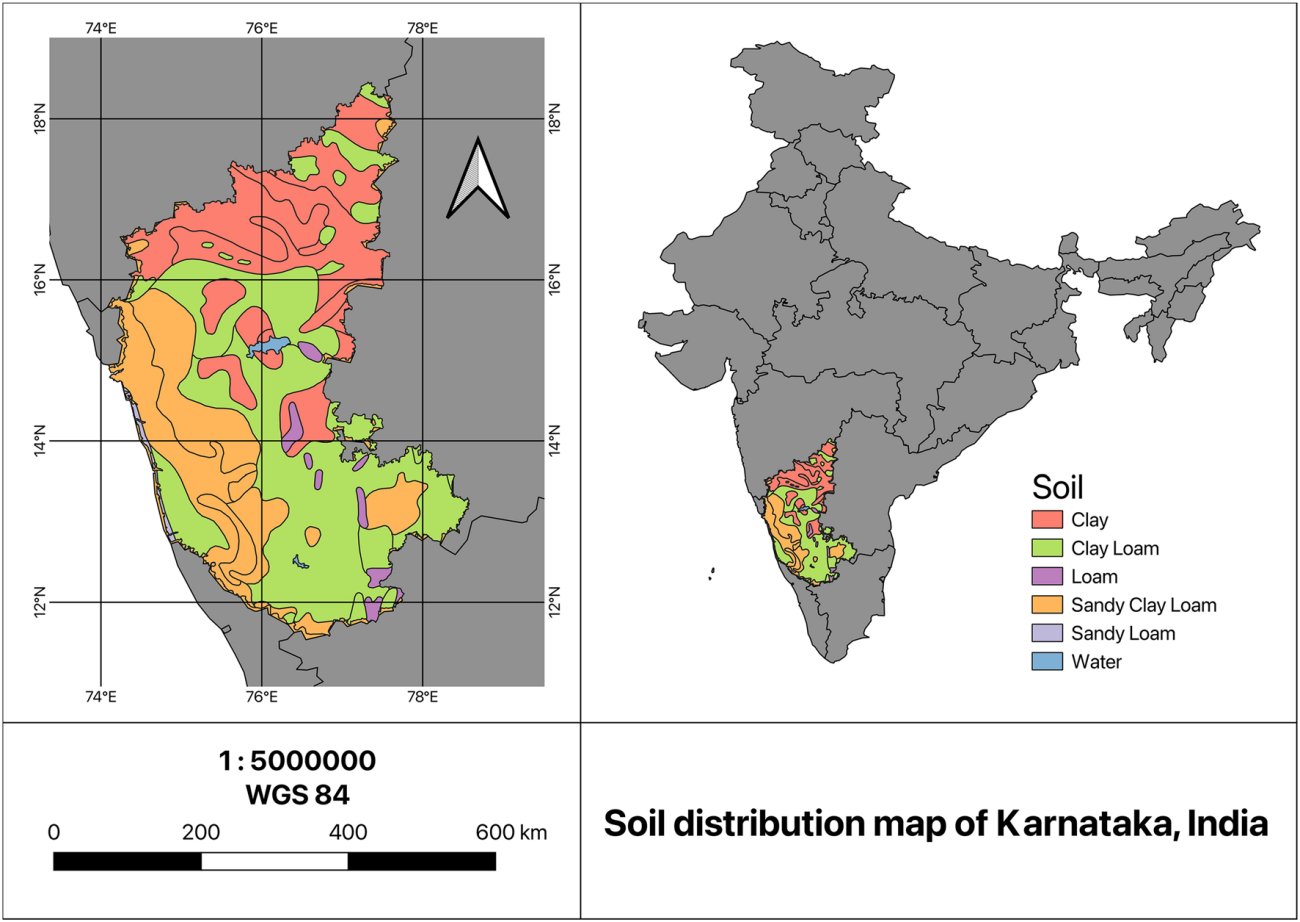
The proposed study area, Karnataka, India and its soil textural types.

The state of Karnataka, situated in the southwestern region of India, is delineated by latitudes 11° 30ʹ N to 18° 30ʹ N and longitudes 74° E to 78° 30ʹ E, based on the World Geodetic System (WGS) 84 datum. The region covers a total land area of 191,791 sq. km, constituting approximately 5.83% of the total area of the country. With a geographical expanse of around 400 by 750 km, Karnataka is positioned in India’s western part of the Deccan peninsular region, where the western and eastern ghats intersect. Karnataka has range of climates owing to diverse topography with a mix of plains, hills, plateaus and coastal areas. The different soil textural types available include *clay, loam, clay loam, sandy loam and sandy clay loam*. Figure 1 depicts the location of Karnataka and its soil distribution.

### Data collection

The initial step in the data collection process involves generating a shapefile from the FAO (Food and Agriculture Organization) web source. A shape file is a format developed by ESRI (Environmental Systems Research Institute, Inc.) that comprises multiple files representing a geographic dataset and its geospatial attributes. It is a standard geospatial vector data that stores information about geographical features such as points, lines, and polygons. The shapefile is imported into QGIS 3.30’s-Hertogenbosch. QGIS is a widely used and reputable open-source software that effectively manages and manipulates geospatial data. It allows users to import, visualise, analyse, and manipulate geographic data in different formats, including shapefiles.

### Pre-processing of data

In the context of soil type analysis, the FAO shapefile contains polygons representing different soil textural types or classes, along with attribute data describing their properties. The imported shapefile is clipped to isolate the specific region of interest, in this case, the Karnataka region. The outline for Karnataka is obtained from DIVA-GIS, a web source that provides country-specific geospatial datasets. The “administrative area” of the geospatial file for India is utilised by importing it into QGIS and using it as a mask layer to clip out the Karnataka region from the world soil map of FAO.

The above process focuses on the analysis of the desired area of interest. The clipped layer corresponding to the Karnataka region is exported as a new layer, providing a distinct dataset tailored to the chosen region. This exported layer is further processed and analysed using geospatial tools and techniques like the *join attributes by location*. The detailed steps involved in data collection are described in Algorithm-01.

**Algorithm-01:** Data collection

Input: The shapefile from FAO

Output: The CSV (comma separated values) file with attributes such as latitude, longitude, region and soil type

Step 1: Obtain the shapefile from the FAO web source.

Step 2: Import the shapefile into QGIS.

Step 3: Clip the layer corresponding to a specific region (Karnataka, India) and export it as a new layer.

Step 4: Extract latitudes and longitudes for the chosen region from the clipped layer using QGIS.

Step 5: Process the exported layer using the geopandas library and export the geo-dataframe as a CSV file.

Step 6: The attribute columns in the dataset include the latitude, longitude, region, and soil type of a specific region.

Step 7: The exported CSV will serve as the dataset for the decision tree algorithms to perform classification.

Using QGIS, an extensive dataset of 160,492 rows is generated using a grid-based approach with a resolution of 0.01 degrees of latitude serving as a geographic unit for the extraction process. This fine-grained grid is superimposed on the shapefile representing the Karnataka region, enabling precise delineation and extraction of data points within each grid cell. Each data point in the extracted dataset corresponds to a specific location defined by latitude and longitude coordinates. Additionally, the dataset includes subregion names, providing contextual information about the particular areas within Karnataka. Moreover, valuable information about soil characteristics is associated with each data point, facilitating subsequent soil type analysis and exploration.

By leveraging the comprehensive dataset derived from the grid overlay technique, a detailed investigation of soil properties at a localised level becomes possible. This enhanced granularity of spatial information within the Karnataka region enables detailed geospatial analysis and exploration of various soil textural types, such as (1) *Clay*, (2) *Loam*, (3) *Clay loam*, (4) *Sandy loam*, (5) *Sandy clay loam* and (6) *Water*. The subregions of Karnataka extracted using QGIS are (1) *Bidar*, (2) *Gulbarga*, (3) *Belgaum*, (4) *Bijapur*, (5) *Bagalkot*, (6) *Raichur*, (7) *Koppal*, (8) *Uttar Kannand*, (9) *Shimoga*, (10) *Udupi*, (11) *Chikmagalur*, (12) *Dakshin Kannad*, (13) *Hassan*, (14) *Kodagu*, (15) *Gadag*, (16) *Dharwad*, (17) *Bellary*, (18) *Haveri*, (19) *Davanagere*, (20) *Chitradurga*, (21) *Tumkur*, (22) *Mysore*, (23) *Mandya*, (24) *Chamrajnagar*, (25) *Bangalore Rural*, (26) *Kolar*, (27) *Bangalore Urban*. Table 1 details the sample of dataset-1 with attributes such as soil and location for the Karnataka region.

**Table 1:** Sample of dataset-1 constituting of statistical characteristics of location and soil for an investigated region.

#	Latitude (N)	Longitude (E)	Soil type	Region in Karnataka
1	17° 42′ 18.432ʹʹ	76° 41′ 47.4972ʹʹ	*Clay*	Bidar
2	17° 26′ 42.432ʹʹ	77° 11′ 47.4972ʹʹ	*Clay loam*	Gulbarga
3	15° 17′ 6.432ʹʹ	76° 39′ 23.508ʹʹ	*Loam*	Bellary
4	13° 58′ 30.432ʹʹ	75° 26′ 11.508ʹʹ	*Sandy clay Loam*	Shimoga
5	14° 17′ 6.432ʹʹ	74° 28′ 35.508ʹʹ	*Sandy loam*	Uttar Kannad

In medicinal plants, accurate data collection plays a pivotal role in understanding their botanical characteristics and conservation status. The IUCN, a membership union of civil society and government organisations, provides a comprehensive and globally recognised list of medicinal plants, including their botanical names and conservation status. It has generated a red list of threatened species that provides extinction risk and species distribution^30^. The red list in IUCN assigns species a ranked threat category, such as (a) Data deficient as DD, (b) Endangered as EN, (c) Least concern as LC, (d) Not evaluated as NE, (e) Near threatened as NT, and (f) Vulnerable as VU through assessment against quantitative criteria based on indicators of extinction risk^16^. Table 2 depicts the sample dataset-2 with medicinal plants, their conservation status and soil type.

**Table 2:** Sample of dataset-2 constituting medicinal plants and soil type for the investigated conservation status.

#	Soil type	Botanical name of medicinal plant	Conservation status
1	*Sandy clay loam*	*Aphanamixis polystachya*	LC
2	*Loam*	*Cinnamomum macrocarpum*	VU
3	*Clay*	*Plectranthus caninus*	LC
4	*Sandy loam*	*Jatropha gossypiifolia*	LC
5	*Loam*	*Zinnia peruviana*	NE
6	*Clay loam*	*Exacum bicolor*	EN
7	*Sandy loam*	*Carica papaya*	DD

### Decision tree algorithm

Of the many types of ML algorithms, supervised and unsupervised learning algorithms are the two main types typically split based on how the machine gathers and learns data. The proposed study showcases the supervised learning algorithms where a stream of codified learning in a database with input and output representation derives the machine experience. The observations in the database, known as samples, consist of features as inputs and target labels as output. The main aim of supervised algorithms is to map the inputs to their respective output with high accuracy. All the supervised algorithms are summarised as shown in Eq. (1), which describes the regularised empirical risk minimisation issue.

1 $${\min}_{f\in H}\frac{1}{n}{\sum }_{i}^{n}Loss\left({y}_{i,}f\left({x}_{i}\right)\right)+\frac{\lambda }{2}{\Vert f\Vert }_{H}.$$

Here, *Loss* denotes the loss function that compares the actual value *y*_*i*_ with the achieved value from the model (*f*) with *x*_*i*_ input variables. The first term measures the quality of fit, and the second term to avoid overfitting in complex models. Decision trees (DT) are a supervised algorithm adopted in this study.

DT employs the sum of squared loss as the empirical risk minimisation and greedy heuristic logic adopted on rectangle regions. It mainly concentrates on classification issues, including continuous and categorical input and output variables^31^. At first, the samples are divided into homogeneous sets considering the features that separate the samples. The nomenclature adopted in DT is root (start of the tree with only outgoing arrows containing all the samples), leaves (terminal node where the decision is complete), nodes, branch, and pruning (removal of a portion of the tree to avoid overfitting). The samples are progressively split, relying on various techniques such as information gain, Gini, reduction in variance, and chi-square^32^.

Entropy quantifies the level of uncertainty associated with the classification of a particular feature. When all elements are exclusively aligned with a singular class, entropy achieves a state of purity, indicating unambiguous and precise classification. The entropy metric ranges between 0 and 1, with 0 representing perfect classification purity, where all elements are distinctly associated with a specified class or a singular prevailing class. Conversely, an entropy value of 1 signifies the maximum level of uncertainty, suggesting a random and indiscriminate distribution of elements across various classes. Mathematically, Entropy is expressed as shown in Eq. (2),

2 $$H\left(S\right)=-\sum_{i=1}^{c}{P}_{i}\cdot {{\text{log}}}_{2}\left({P}_{i}\right),$$

where *S*—set of data points, *c*—no. of classes, *P*_*i*_—probability.

The Gini Index, alternatively termed Gini impurity, quantifies the likelihood of misclassification for a given feature chosen at random. It attains a state of purity when all elements are exclusively associated with a singular class, signifying flawless classification. Analogous to entropy, the Gini index spans the numerical range between 0 and 1. A value of 0 signifies impeccable classification purity, where all elements are assigned to a specified class or a singular class prevails. Conversely, a value of 1 denotes a haphazard distribution of elements among diverse classes. A Gini Index value of 0.5 indicates an equitable distribution of elements across multiple classes. Mathematically, Gini Index is expressed as shown in Eq. (3),

3 $$Gini\, index=1- \sum_{i=1}^{c}{\left({P}_{i}\right)}^{2},$$

where *c*—no. of classes and *P*_*i*_—probability.

The tree-based algorithms adopt the ensemble technique to improve the accuracy parameter. The ensemble method combines weak learners to attain strong, high-performance learners^33^. Bagging and boosting are the two types of techniques in ensemble learning. Bagging combines multiple classifier results for a subsample series of the same primary dataset. Various sub-datasets are created by randomly selecting the samples and repeating them. A model is created for every sub-dataset, and the final prediction is generated by calculating the mean, mode, or median of all the model responses. The obtained results are more robust than the primary ones. The set of techniques under bagging are random forest and extra trees algorithm.

Boosting technique converts weak learners to strong learners. The final prediction combines other predictions using the weighted average approach. The critical difference from bagging is that the learners are built sequentially. In bagging, every tree made is independent, whereas in boosting, the trees are constructed considering the misclassified samples, and weights are redistributed considering the difficult cases in the further subsets. Boosting technique examples are Ada boost and XGBoost.

#### Random forest

The DT algorithms, such as extra trees classifier, random forest (RF), and XGBoost, are ML techniques that emerge from ensemble learning methods. RF is a fusion of decision trees where each tree votes for the most recurrent band values of the input vector to perform classification. The predictive trees are grown randomly from the input dataset by bagging or boosting. The input dataset is split using measures such as the Gini index or information gain that helps to maximise the dissimilarity between the class labels and thus define the best split by creating subsets. More details of RF can be fetched in Refs.^34,35^.

#### Extra trees classifier

Extra tree classifier (EXTC)^36^, also known as an extremely randomised tree, constructs independent decision trees for classification and includes robust randomisation methods to lower the variance of the classification model. The critical differences between RF and EXTC are: (a) EXTC utilises the complete training set to build each tree, whereas RF applies the bagging technique. (b) Nodes (variable and variable index) are chosen randomly in EXTC. In contrast, RF best split is performed by optimising the variable and variable index from a random subset of variables, which makes the trees uncorrelated and diversified.

EXTC offers a distinct advantage in terms of computational efficiency compared to RF. Its randomisation approach enables the construction of decision trees at a faster pace compared to RF. While RF evaluates numerous potential splits to determine the optimal one, consuming substantial computational resources, EXTC employs random splits without exhaustive evaluation, leading to quicker training times. This speed benefit renders EXTC particularly suitable for handling extensive datasets or scenarios where computational efficiency takes precedence.

#### XGBoost

Extreme gradient boosting (XGBoost)^37^, a popular ML algorithm based on the DT technique, creates a forest containing many DTs. This technique uses the series dataset training procedure to integrate the weaker predictors and accomplish powerful predictors. The overfitting issue is alleviated by introducing the regularisation term during the modelling procedure. It is an ensemble technique that trains successively to optimise the objective function till it reaches the lowest value (training halts).

#### Bagging classifier

Bootstrap AGGregatING, known as bagging, is an ensemble technique that trains the classifiers randomly on the original training set *T* = *t*_1_*,t*_2_*,…t*_*n*_, where it represents the *i*th sample and *n*—total samples. The technique aggregates every prediction to the final prediction by adopting the method of averaging or voting. The bagging classifier reduces the variance of the model and thus reduces the overfitting issue. The bagging classifier ensemble can be represented as showed in Eq. (4),

4 $${\widehat{f}}_{bag}\left(x\right)=\frac{1}{B}{\widehat{f}}_{\left(b\right)}\left(x\right),$$

where ${\widehat{f}}_{bag}$—the aggregated or ensemble prediction for the input *x*, *B* is the number of base classifiers and ${\widehat{f}}_{\left(b\right)}\left(x\right)$—the prediction of the *b*th base classifier for the input *x*.

## Proposed methodology

**Figure 2:**
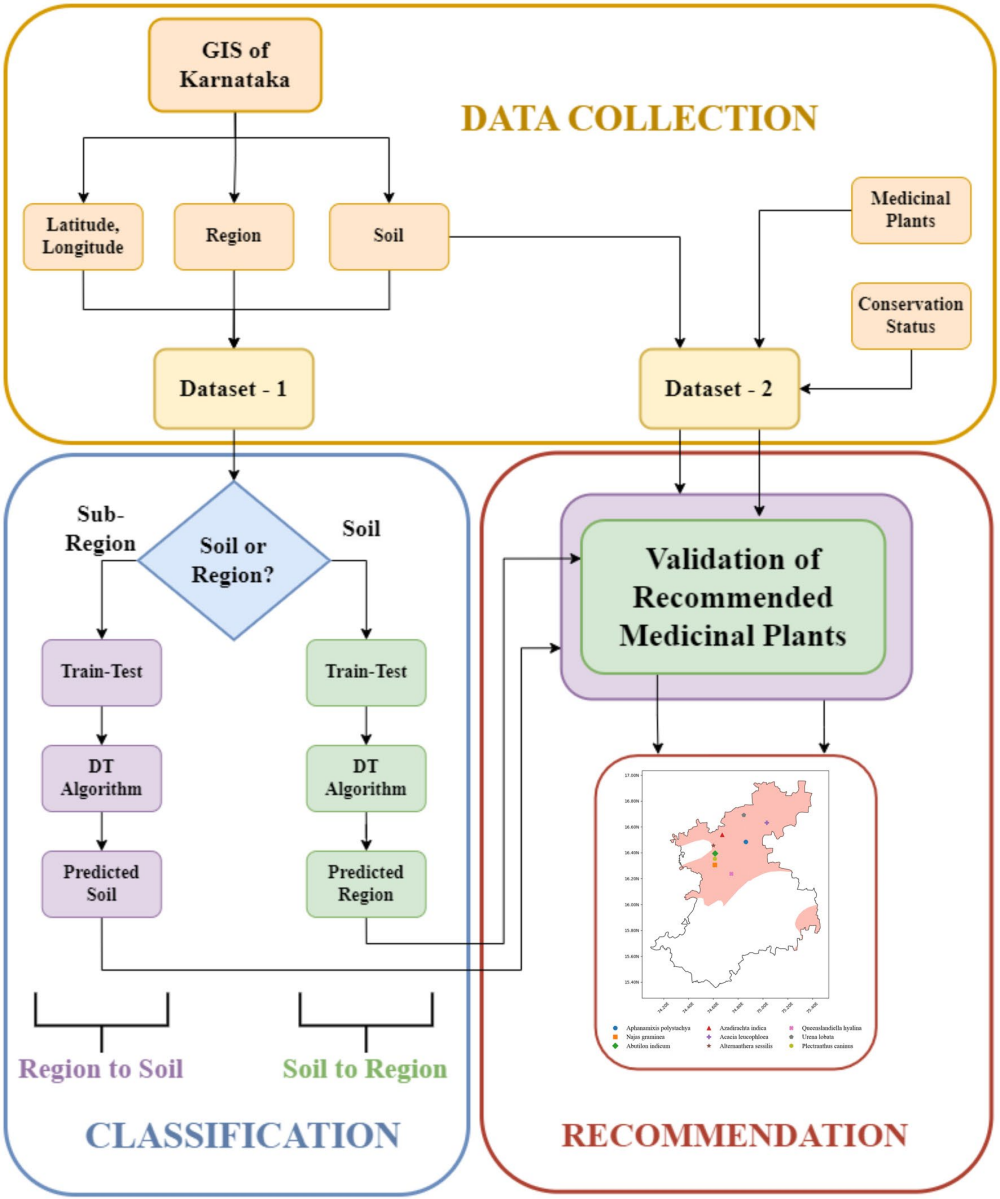
The proposed approach *for GeoHerb.*

The main objective is to recommend vulnerable medicinal plants and their potential growing spots to conserve them using the soil and GIS data for the Karnataka region. The approach considered the 27 subregions and six soil textural types of the Karnataka region, where users can select either the soil type or the area of interest for which the proposed system recommends the potential medicinal plants that can be grown efficiently. The suggested approach consists of three phases: data collection, classification, and recommendation. The data collection phase of the proposed method includes two different stages, dataset-1 with attributes such as latitude, longitude, subregions, and their corresponding soils and dataset-2 with attributes such as vulnerable medicinal plant botanical names, their conservation status and suitable soil textural types. Figure 2 shows the detailed flow of the approach adopted to build the proposed system (*GeoHerb*). In the classification phase, based on the user input (soil type or region), the ML model is developed to classify the respective output (for soil type as input, the target label is its subregion, and for subregion as input, the target is the suitable soil type). Hence, two ML models are designed to predict the output labels. Apart from decision tree-based models, an alternative approach adopted is the kNN classification algorithm. The algorithm consists of applying Euclidean distance to measure the feature point distance. The algorithm shows different behaviour based on the k (nearest neighbour) value. The default parameter for k = 5 is used in the work.

In the third phase (recommendation), the recommended plant species, derived from the classification phase, are seamlessly integrated into a choropleth map, a geospatial visualisation technique that employs colour-coded areas to depict the spatial distribution patterns of the predicted soil textural types and their associated plant communities. This geo-visualisation technique leverages thematic shading and colour gradients, allowing for the visualisation of plant abundance, species richness, and ecological zonation within the region of interest. By interacting with the map, users can access detailed botanical information by clicking on plant icons. This enables an enhanced comprehension of the intricate relationships between soil characteristics, plant suitability, and geographical context. The choropleth map is a valuable tool in exploring and analysing plant-environment interactions, facilitating informed decision-making in ecological management and land-use planning endeavours.

The research explores the challenge of creating novel datasets by curating attributes such as soil, GIS information, medicinal plant botanical names and conservation status that contribute at a high rate to the growth of medicinal plants. The medicinal plants considered in the research are vulnerable, as per the IUCN records. The developed system with ML models such as random forest, XGBoost, kNN, bagging classifier and extra trees classifier predict the medicinal plants suitable to grow efficiently to the specific soil or region with a high accuracy rate. The proposed extra trees classifier showcases a higher accuracy rate than other proposed models.

The work intends to serve society to overcome the issue of vulnerable medicinal plants at extinction and assist the public and its stakeholders to a great extent in increasing their growth.

## Results and discussion

**Figure 3:**
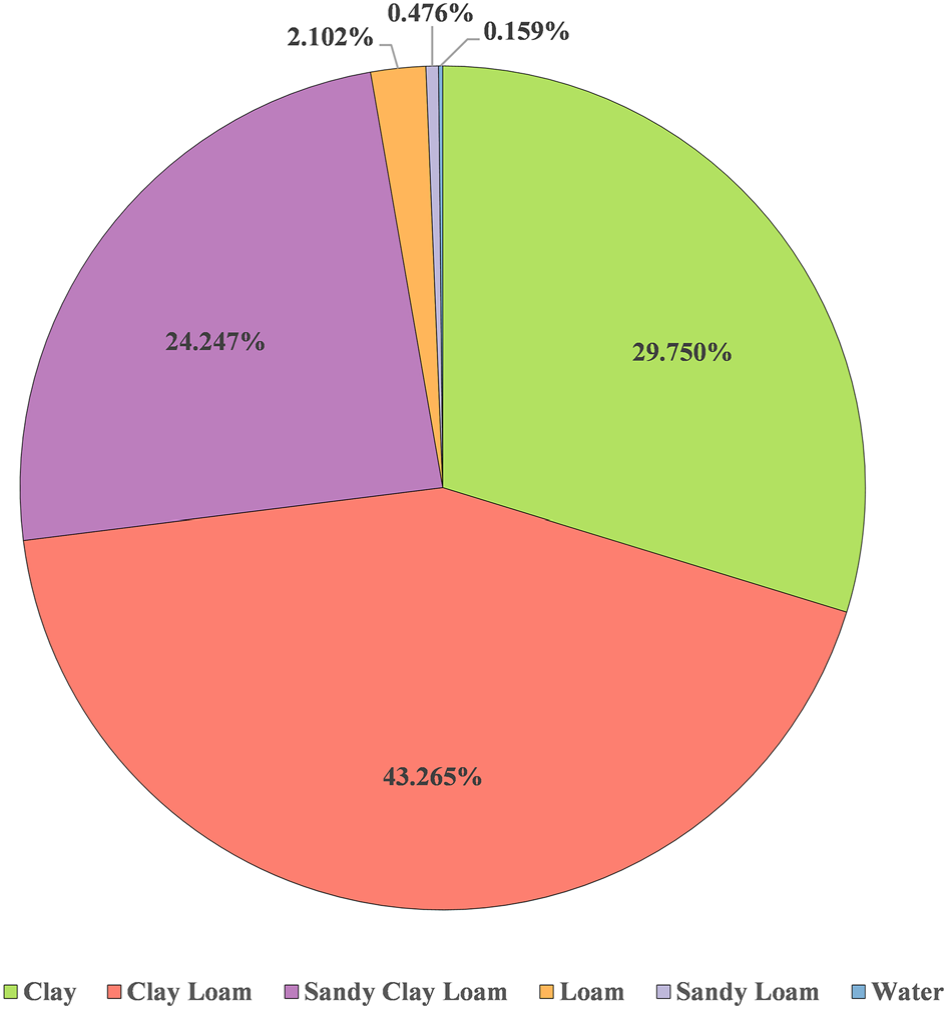
The percentage distribution of six types of soil for Karnataka region.

**Figure 4:**
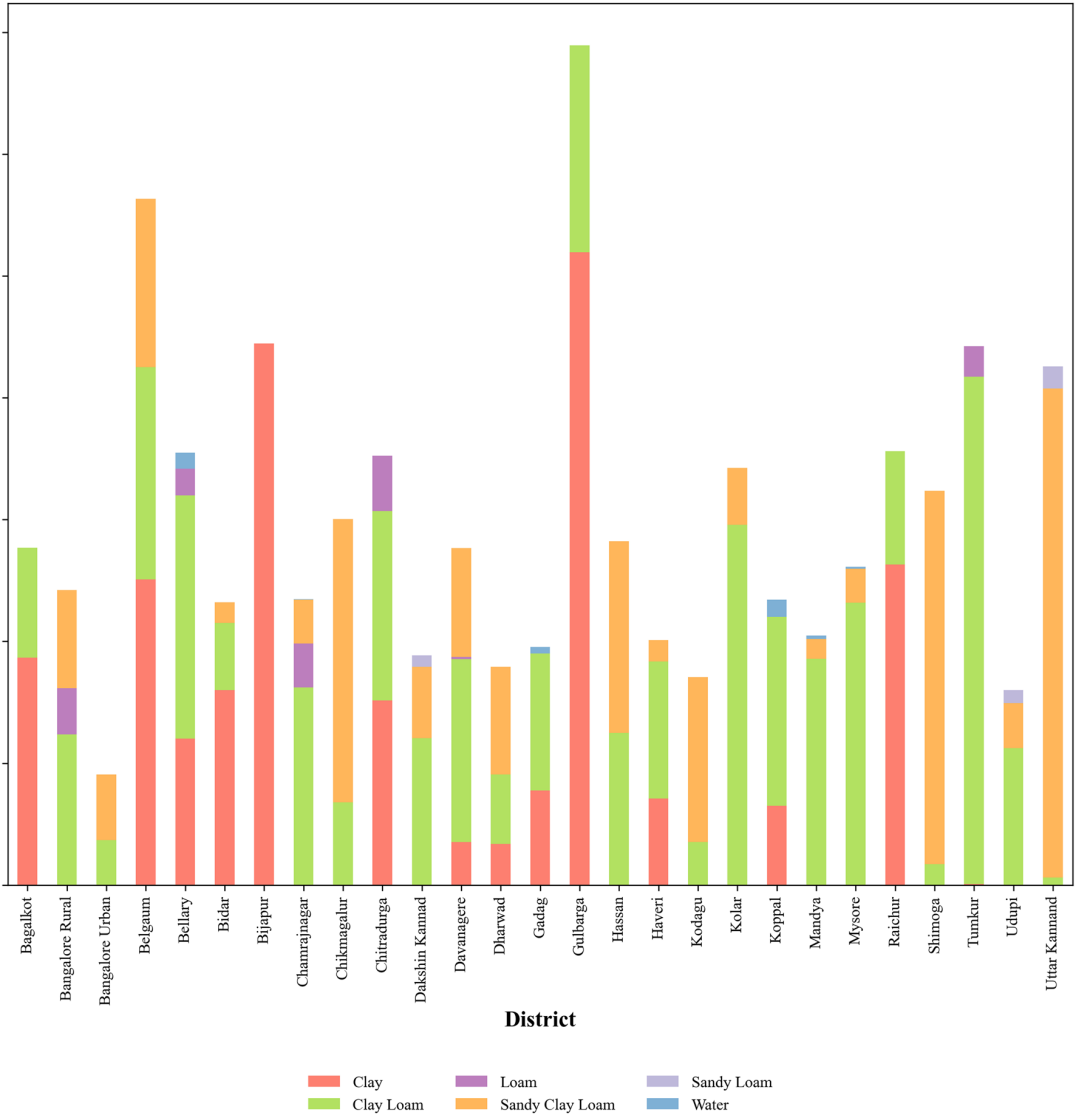
The distribution of soil for the twenty-seven subregions of Karnataka region.

**Figure 5:**
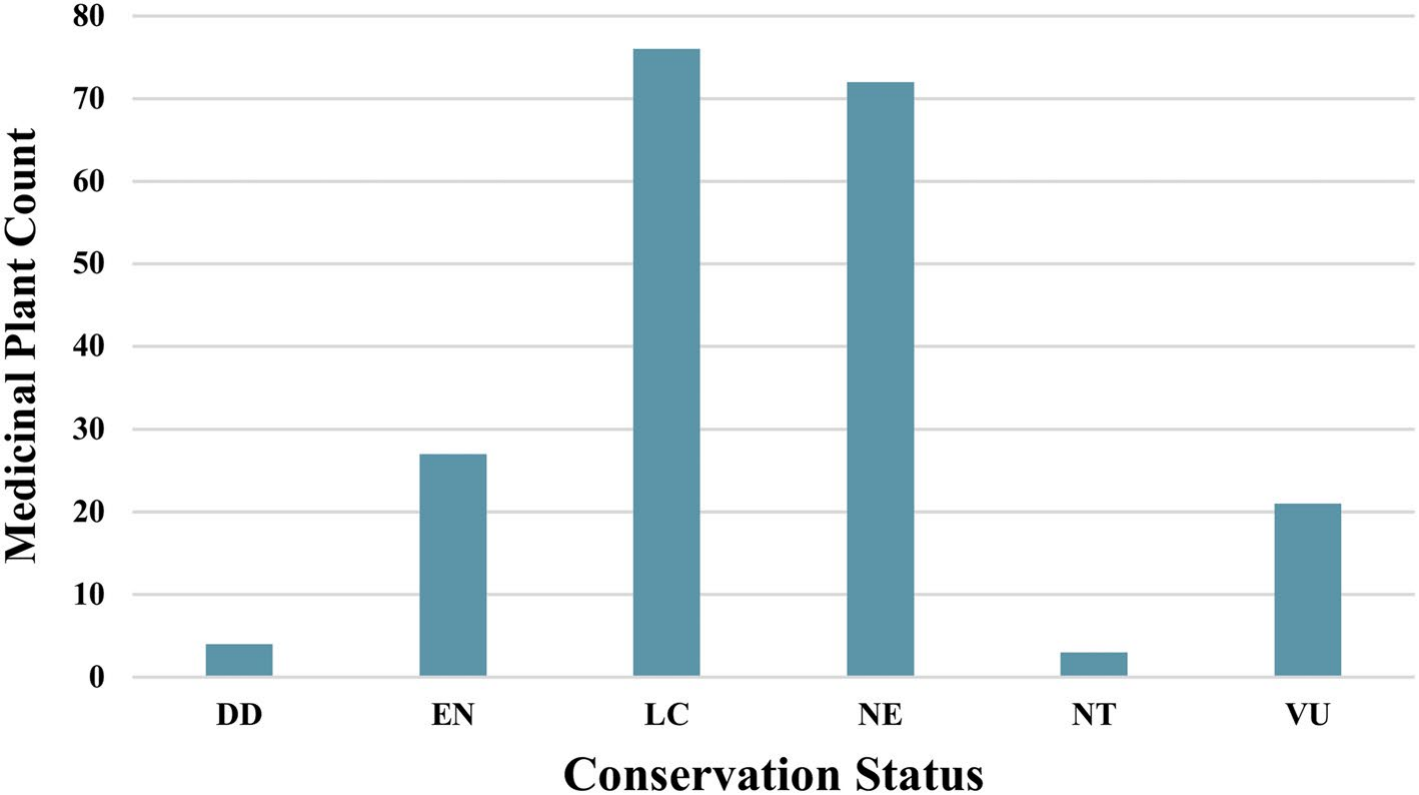
Distribution of medicinal plants by their conservation status for the region of Karnataka, India.

This proposed approach aims to predict, (a) the soil type for the given subregion as input and (b) the subregion well suited for the given soil type input. The predicted values are shown on the map to showcase the suitable medicinal plants of that region or soil type to enhance its growth and development. The research employed QGIS to extract geospatial data from soil maps, facilitating the creation of dataset-1 and dataset-2. Figure 3 clearly details the percentage of various soil type distributions available in the region of Karnataka. Similarly, Fig. 4 showcases the distribution of soil textural types for the different subregions of Karnataka. The distribution of 150 medicinal plants considered in dataset-2 and their respective conservation status drawn from IUCN is shown in Fig. 5. It is evident that many herbs are endangered, not evaluated, or vulnerable. Hence, herb conservation is an immediate need of the hour.

The proposed prediction involves the implementation of many classifiers such as RF, EXTC, bagging, XGBoost and kNN. Every classifier algorithm possesses a set of parameters to be tuned to yield good prediction performance. Some of the parameters tuned include (a) RF and EXTC classifier: *n estimators* = 100; *criterion* = gini; *min samples split* = 2, and *min samples leaf* = 1. (b) XGBoost: *booster* = gbtree, *learning rate* = 0.3, *gamma* = 0, *max depth* = 6, and *min child weight* = 1. (c) Bagging Classifier: *n estimators* = 10, *max samples* = 1.0, *max features* = 1.0. The best model is selected based on the performance metrics. Table 3 displays the performance metrics of classification models, offering insights into their accuracy metric in categorising soil textural types within specific subregions. Similarly, Table 4 showcases the performance metrics of classification models, providing valuable information on their accuracy in classifying subregions for soil textural types. Tables 3 and 4 show values for the dataset split in the ratio of 80:20 (train set—128,393 rows and test set—32,099 rows) and serve as an essential reference, highlighting the effectiveness of the models in categorising.

**Table 3:** Performance metrics for the classification of soil type for the given subregion.

Model	F1 score (%)	Precision (%)	Recall (%)	Accuracy (%)	
				Training	Testing
Random forest	98.74	98.77	98.70	100	98.27
EXTC	**98.77**	**98.83**	**98.71**	**100**	**99.01**
XGBoost	97.83	97.66	97.99	98.57	97.44
kNN	98.86	98.87	98.85	99.69	97.58

Significant values are in bold.

**Table 4:** Performance metrics for the classification of subregions for the given soil type.

Model	F1 score (%)	Precision (%)	Recall (%)	Accuracy train (%)	Accuracy test (%)
Random forest	98.53	98.53	98.53	100	98.05
EXTC	**98.63**	**98.63**	**98.63**	**100**	**98.76**
Bagging	98.54	98.54	98.54	99.89	97.94
kNN	98.62	98.62	98.62	99.48	97.29

Significant values are in bold.

In evaluating the classification models presented in Tables 3 and 4, it is noteworthy to highlight that different parameters are utilised for all models, underscoring the remarkable performance achieved in classifying the soil type and subregion, respectively. The different parameter configuration reflects the model’s intrinsic capabilities, devoid of manual tuning or customisation. Despite this, the attained performance metrics, such as f1-score, precision, accuracy and recall, exhibit noteworthy rates and effectiveness in categorising soil textural types within subregions and vice-versa.

The top-performed models are highlighted and it is evident from Table 4 that XGBoost is not utilised to classify subregions based on given soil textural types due to its subpar performance in the initial evaluation, as its results failed to exhibit the desired level of accuracy and effectiveness compared to other models. In contrast, the bagging classifier is not employed in the soil type classification within subregions shown in Table 3 due to its limitation of not supporting multiple target columns (soil textural types). The above result showcases that EXTC and RF are selected to ensure accurate and reliable classification compared to other models.

However, the kNN algorithm demonstrated competitive accuracy in both classifications of decision tree-based models. In particular, the EXTC classifier outperformed all other models in its cross-validation scores. The high accuracy attained consistently by EXTC suggests its effectiveness in capturing and utilising the underlying patterns and variations in the dataset across different folds of the data. Hence, proceeded with EXTC as the top performer when compared to other classifiers for the current research predictions.

**Figure 6:**
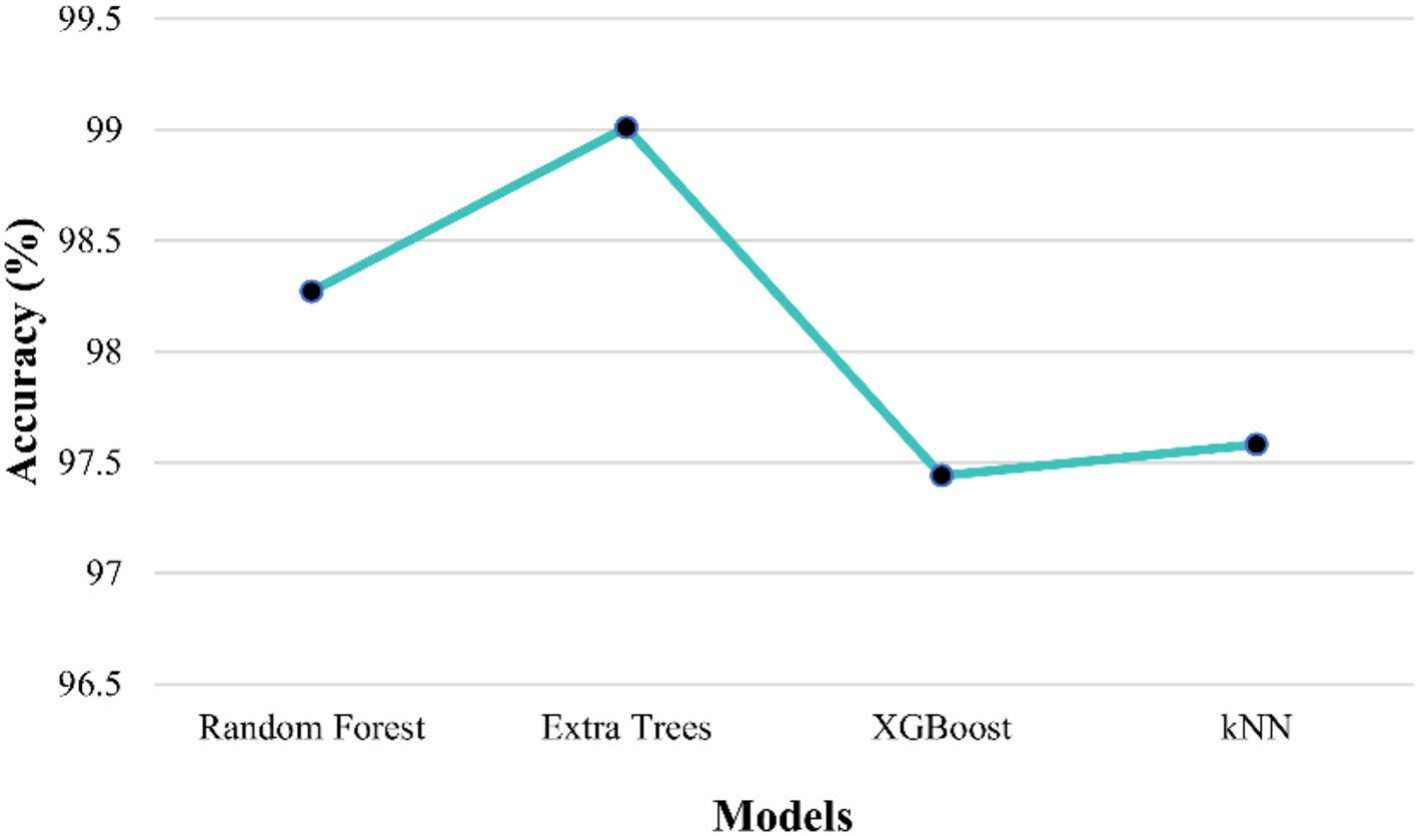
Comparison of testing accuracy rate of EXTC with other models for the classification of soil classes.

**Figure 7:**
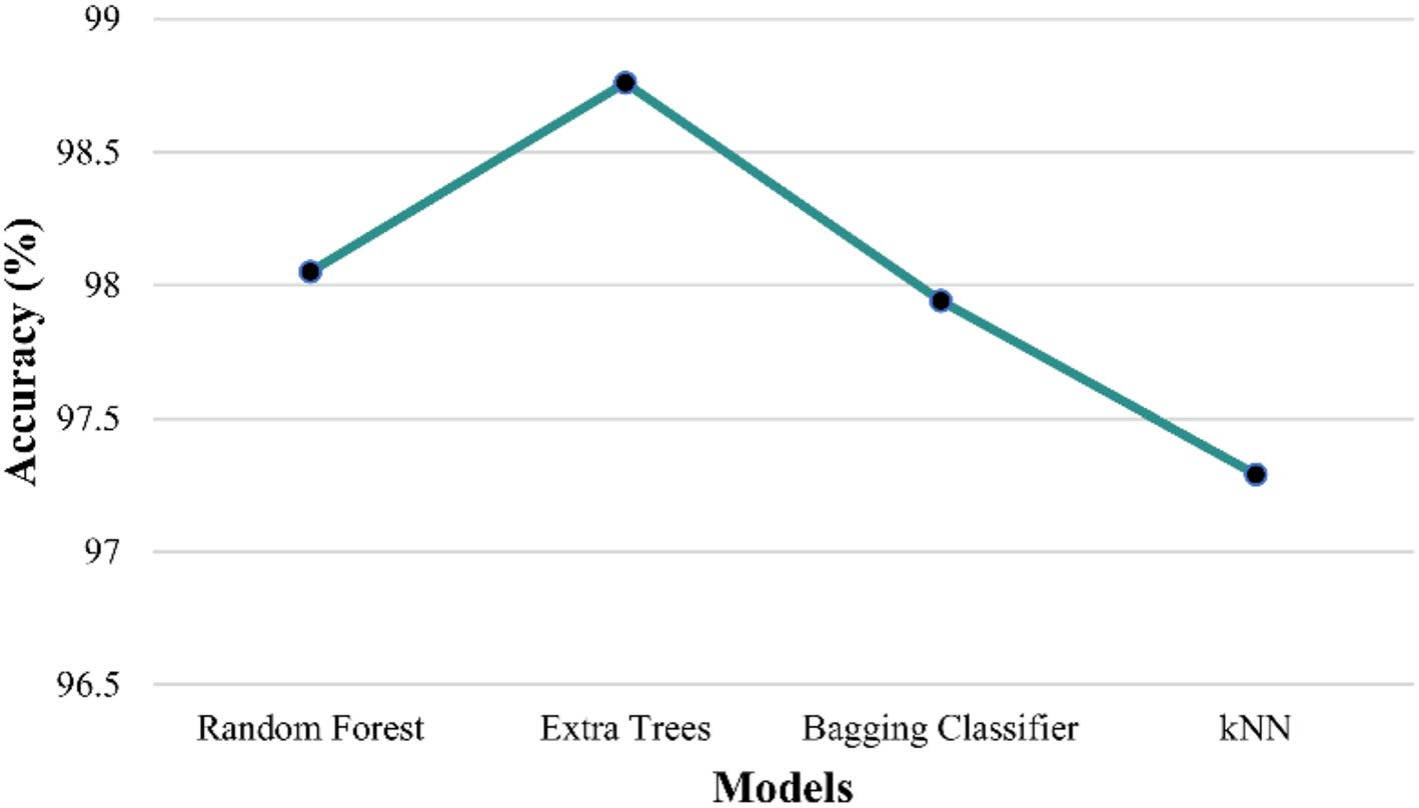
Comparison of testing accuracy rate of EXTC with other models for the classification of subregion classes.

The results in Figs. 6 and 7 highlight the robustness and reliability of the EXTC model for the classification of soil and subregion respectively. The adopted cross-validation process utilised the k-fold value of 5, for which the EXTC showed consistent top accuracy of 99.01% for classifying soil textural types within subregions and 98.76% for categorising subregions based on soil textural types. The robustness demonstrated by the cross-validation scores further supports the notion that the examined classification models can efficiently classify soil textural types within subregions and vice-versa, making them valuable tools in geospatial analysis, environmental studies, and related domains.

The research concludes by utilising the two datasets, the ML model with the EXTC classifier algorithm developed to recommend the most suitable medicinal plant based on the user-selected soil type or subregion. It is determined through a rigorous evaluation that EXTC and RF classifiers emerged as the best-performing models. As EXTC consistently exhibits high accuracy across various metrics, highlighting its robustness and reliability in classification, it is chosen as the best model in the work. A choropleth visualisation provides a comprehensive understanding of the results, enabling an intuitive and informative representation of the outcomes in maps with meaningful colour representation for soil and herbs.

**Figure 8:**
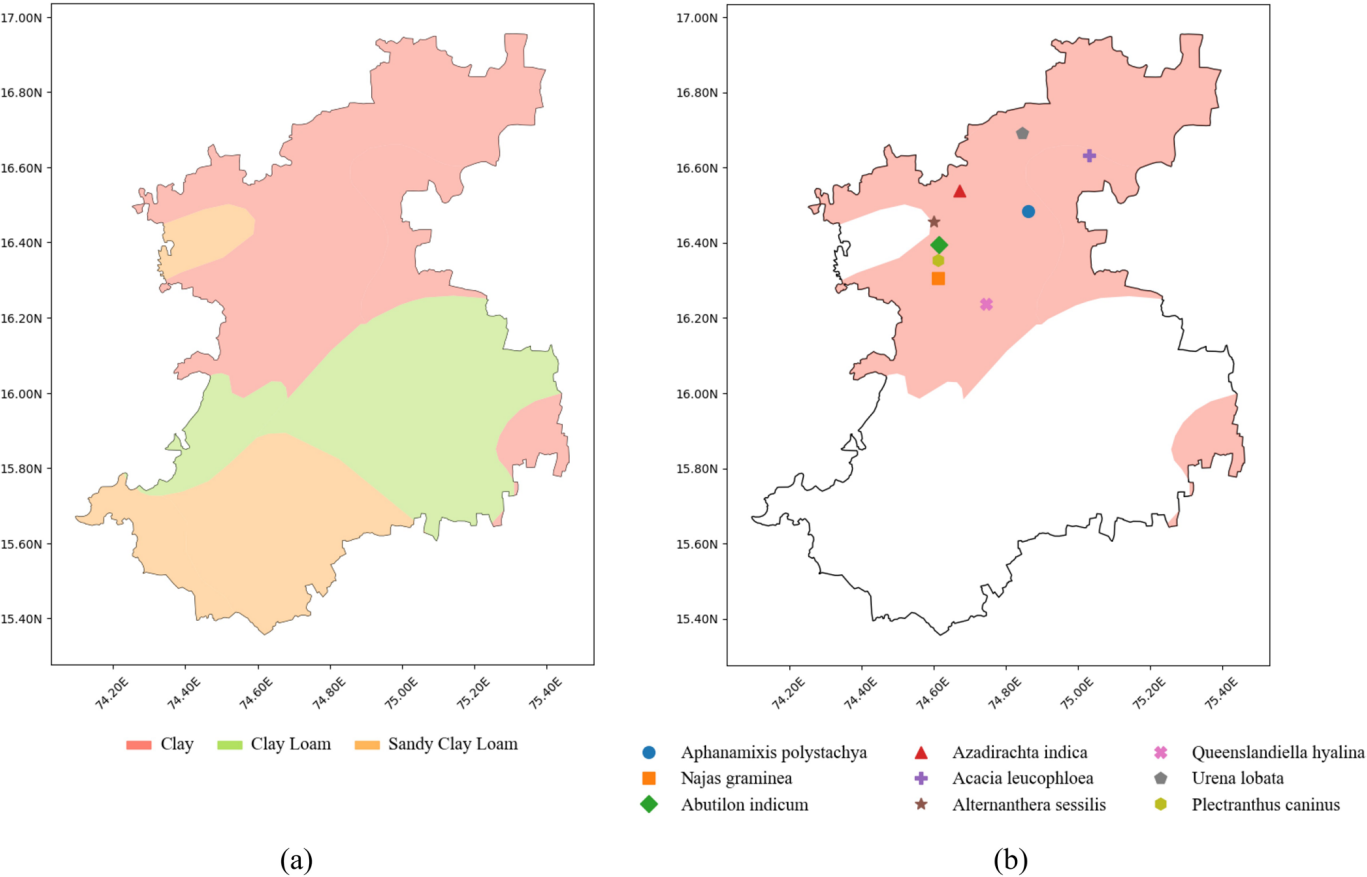
(**a**) Distribution of soil in *Belgaum* subregion. (**b**) Plot of LC herbs in *Belgaum* subregion.

Figure 8 showcases a collage of plots that offer valuable insights drawn from different experiments carried over to derive the performance of the EXTC model over the distribution of soil textural types within specific subregions selected by the user and the corresponding medicinal plant’s recommendation based on their conservation status. The plot in Fig. 8a illustrates the soil distribution within the user-selected subregion (*Belgaum*), highlighting its different soil textural types and spatial extent. Figure 8b complements the above information by indicating the recommended medicinal plants with conservation status as LC that thrive in clay soil within the *Belgaum* subregion.

**Figure 9:**
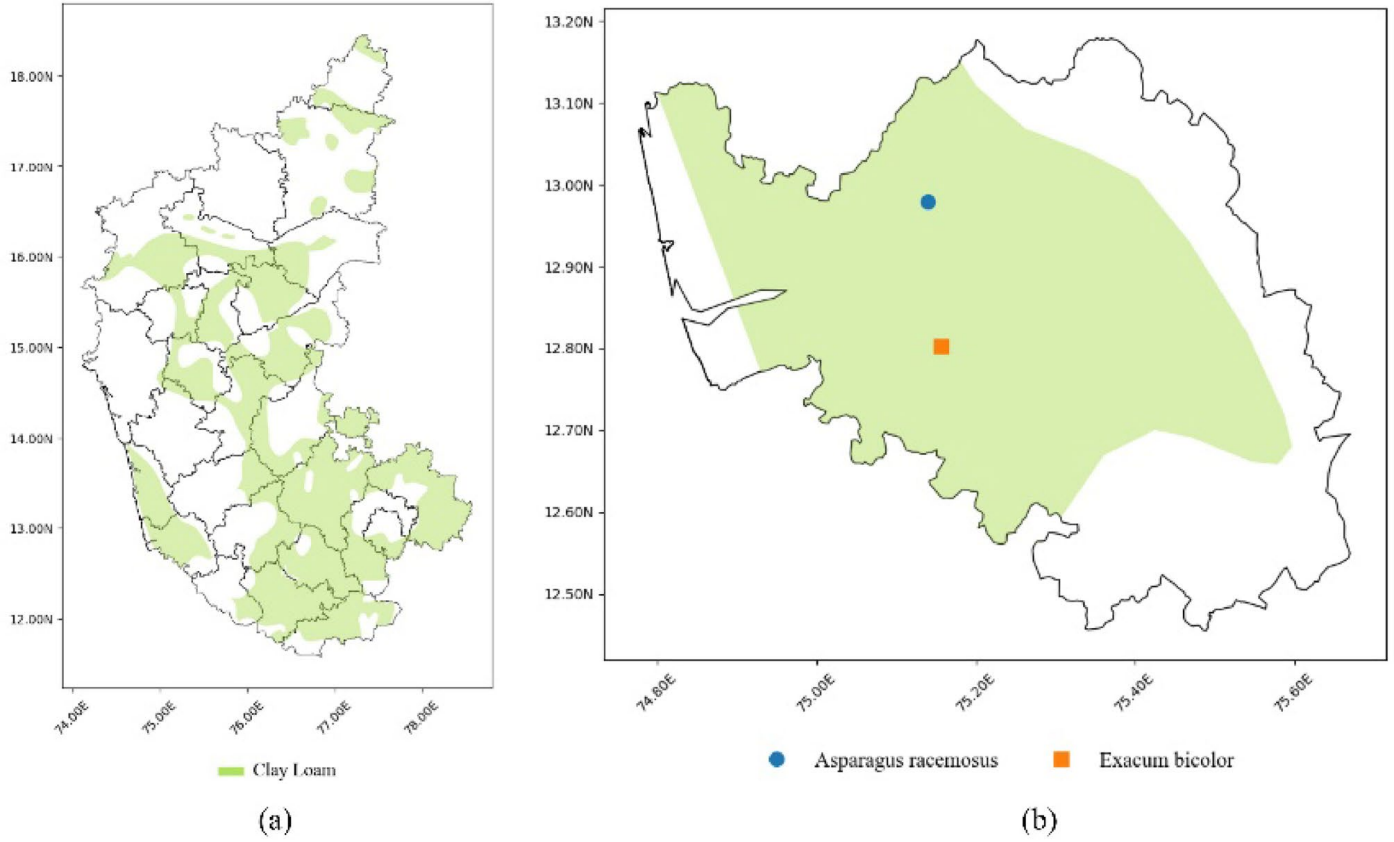
(**a**) Distribution of *clay loam* soil in Karnataka. (**b**) Plot of EN herbs, potentially growable in *Dakshin Kannad* subregion.

Figure 9 details the recommendation of medicinal plants for the user-selected soil type (*clay loam*) in the Karnataka region. The plot in Fig. 9a illustrates the distribution of clay loam soil within Karnataka. Figure 9b complements the above information by indicating the recommendation of medicinal plants with conservation status as EN that thrive in *clay loam* soil within the *Dakshin Kannad* subregion.

From the misclassification results detailed in Tables 5 and 6, the following are the observations as the EXTC model overcomes the overfitting issue and generalises to a high rate. The misclassification noticed in soil classification is at the border of the two soil textural types. For example, the scattered soil textural types in the *Belgaum* region are *clay, clay loam and sandy clay loam* as witnessed in Fig. 8a. Similarly, the few misclassified results observed in Table 6 are due to the subregions that share a common border. For example, the scattered distribution of *clay loam* soil in the Karnataka region is witnessed in Fig. 9a. These misclassifications are insignificant as the potential medicinal herbs pertaining to specific soil or region are displayed using a choropleth map to the end users.

**Table 5:** The analysis of misclassification for the soil classification.

#	Soil type	Total samples	# Correct classification	# Incorrect classification	Error (%)
0	*Clay*	9396	9314	82	0.87
1	*Clay loam*	13,849	13,676	173	1.25
2	*Loam*	665	637	28	4.21
3	*Sandy clay loam*	7895	7843	52	0.66
4	*Sandy loam*	145	134	11	7.59
5	*Water*	149	143	6	4.03
	Total	31,950	31,589	361	3

**Table 6:** The analysis of misclassification for the subregion classification.

#	Sub regions	# Samples	# Correct classification	# Incorrect classification	Error (%)
1	*Bagalkot*	1081	1067	14	1.30
2	*Bangalore Rural*	937	910	27	2.88
3	*Bangalore Urban*	360	346	14	3.89
4	*Belgaum*	2252	2234	18	0.80
5	*Bellary*	1435	1420	15	1.05
6	*Bidar*	916	910	6	0.66
7	*Bijapur*	1693	1682	11	0.65
8	*Chamrajnagar*	947	939	8	0.84
9	*Chikmagalur*	1252	1230	22	1.76
10	*Chitradurga*	1388	1374	14	1.01
11	*Dakshin Kannad*	746	736	10	1.34
12	*Davanagere*	1027	1003	24	2.34
13	*Dharwad*	729	717	12	1.65
14	*Gadag*	806	789	17	2.11
15	*Gulbarga*	2734	2721	13	0.48
16	*Hassan*	1184	1161	23	1.94
17	*Haveri*	793	772	21	2.65
18	*Kodagu*	693	681	12	1.73
19	*Kolar*	1406	1396	10	0.71
20	*Koppal*	975	953	22	2.26
21	*Mandya*	820	805	15	1.83
22	*Mysore*	1047	1032	15	1.43
23	*Raichur*	1422	1407	15	1.05
24	*Shimoga*	1295	1267	28	2.16
25	*Tumkur*	1811	1786	25	1.38
26	*Udupi*	678	670	8	1.18
27	*Uttar Kannad*	1672	1659	13	0.78
	Total	32,099	31,684	415	1.5

The above results clearly state that the approach designed in the current research provides ample information about the medicinal plants of specific regions or soil of interest. Hence, the model promises to promote the growth of the vulnerable herbs that are silently disappearing. The spread of the above knowledge is one of the best approaches to hit the stakeholders of medicinal herbs.

Table 7 compares the proposed system results with the related works. It clearly depicts that the proposed EXTC model demonstrates better accuracy when compared to other related works for soil classification. The limitation of the GeoHerb is not considering the climatic conditions of the region to predict the herb species. All the findings of the work demonstrate the potential aspect of amalgamating the geospatial analysis and machine learning techniques to support the decision-making processes related to the recommendation of exposed medicinal plants from its soil and region classification.

**Table 7:** Comparison of proposed system with related works on soil classification.

#	References	Method	Accuracy (%)
1	Aydın et al.^38^	Decision tree classifier (CART)	90.66
2	Azmin et al.^39^	Random forest	97.23
3	Proposed system	EXTC	99.01

## Conclusion

The research explores the possibilities of predicting the titles of vulnerable medicinal plants in certain regions using GIS and machine learning techniques. The prediction aims to conserve medicinal plants and help in their growth as they are the main source to cure diseases and assist in new drug discovery. The QGIS tool derives the GIS and soil information from the FAO web source. The dataset on vulnerable herbs and their conservation status is extracted from the IUCN records. The methodology is applied to Karnataka, the southern region of India, specifically chosen as the case study. The region consists of 27 subregions and six different soil textural types.

Through the GeoHerb-designed system, the user is given the option to provide either the soil type or subregion of interest as input to predict the potential medicinal herbs (vulnerable) that can be grown efficiently. Few decision tree techniques are employed along with simple classification algorithms such as random forest, extra trees classifier, bagging classifier, XGBoost and kNN. The proposed datasets comprise 160,492 rows of locations with soil information and 150 herbs with their conservation status. The five ML model trials on the dataset showcased competitive accuracy rates. In particular, the extra trees classifier presented 98.76% and 99.01% accuracy for both (prediction of subregion labels and soil type labels, respectively) models.

The convergence of GIS and soil analysis guided by machine learning algorithm revolutionize the understanding, cultivating needs and sustainable utilization of vulnerable medicinal plants. The projected design is the state-of-the-art approach as it bridges the gap between modern techniques and traditional approaches that foster the betterment of society and healthcare practices. The adopted method provides a rapid analysis of the medicinal plants through maps. Many of its stakeholders benefit to a large extent as many herbs are economical products of the country. The work can be extended to implement other learning algorithms in machine learning and apply GIS on different countries contributing to the growth of vulnerable herbs, and production of general crops and extending to add many layers in QGIS to extract the climate and geo-location information suitable for the herbs.

## Data Availability

The datasets used and/or analysed during the current study available from the corresponding author on reasonable request.
